# Peripheral Nerve Stimulator Versus Ultrasound-Guided Femoral Nerve Block for Knee Arthroscopy Procedures: A Randomized Controlled Trial

**DOI:** 10.7759/cureus.32043

**Published:** 2022-11-30

**Authors:** Sanjaya K Behera, Bharat Gunupuru, Lingaraj Sahu, Saswati Das

**Affiliations:** 1 Anaesthesiology and Critical Care, Kalinga Institute of Medical Sciences, Bhubaneswar, IND; 2 Anaesthesiology, Maharaja Institute of Medical Sciences, Vizianagaram, IND

**Keywords:** femoral nerve block, patient satisfaction, duration of block, regional anesthesia, postoperative pain relief

## Abstract

Background

Femoral nerve block (FNB) provides effective analgesia and is a widely used technique for postoperative pain relief for orthopedic procedures on lower limbs. This study aims to compare the efficacy of ultrasonography (USG) versus peripheral nerve stimulator (PNS)-guided FNB in knee arthroscopic procedures.

Methodology

This randomized comparative study included two study groups with 30 participants in each group who were given FNB with either PNS or USG for knee arthroscopic procedures following spinal anesthesia. The study evaluated the number of needle repositioning, the time taken for performing the block, the efficacy of postoperative analgesia based on the duration of the block, and patient satisfaction.

Results

The number of needle repositioning and time taken to finish the procedure using USG was lower compared to the group using PNS (p < 0.001). The duration of the block was comparable in both groups (p = 0.584). Patients were satisfied with both techniques and responded as either very good or outstanding and chose neither as inferior (p = 0.310).

Conclusions

Both techniques have equal efficacy concerning the duration of the effect of the block and patient satisfaction. However, the procedural time and number of needle repositioning were significantly less in the group where USG was used for the block.

## Introduction

Regional anesthesia is the preferred anesthetic technique for lower limb orthopedic procedures as it provides excellent perioperative pain relief, reduces systemic analgesic requirements, and allows early ambulation, thereby decreasing the chances of deep vein thrombosis [1].

Arthroscopic knee surgery has evolved to encompass several surgical interventions of the knee and is associated with severe postoperative pain, which can contribute to immobility-associated complications, delayed hospital discharge, and increased morbidity [2]. Although there are multiple techniques of regional anesthesia that provide good sensory block while minimizing the motor block for better postoperative mobility, the femoral nerve block (FNB) is an easy block with good results. FNB administered using the landmark technique, peripheral nerve stimulation (PNS), or ultrasonography (USG) provides effective analgesia by side-stepping the side effects associated with central neuraxial blockade [3]. The methods to establish the position of the nerve are either guided by elicitation of paresthesia or identification of an appropriate motor response on nerve stimulation. However, the sensitivity for the detection of needle-to-nerve contact in both techniques is low [4].

USG-guided blocks have revolutionized the use of blocks in clinical practice as they offer the added advantage of visualizing the spread of the local anesthetic solution around the targeted nerves [5,6]. Nonetheless, only a few studies have compared USG-guided blocks with electrical PNS, and the potential advantages of USG, such as shortened block performance time and/or reduced number of needle passes, the occurrence of paraesthesia, and the frequency of accidental vascular punctures, must be further evaluated.

## Materials and methods

After obtaining due permission from the Hospital Ethics Committee (KIMS/KIIT/IEC/150/2018), 60 patients posted for knee arthroscopy surgeries at Pradyumna Bal Memorial Hospital, Kalinga Institute of Medical Sciences, Bhubaneswar from September 2019 to July 2021 were assessed for the inclusion and exclusion criteria and were included in this prospective, interventional, randomized study. The trial was registered with the Clinical Trials Registry India (REF/2019/07/027059). The inclusion criteria comprised patients aged 16 to 65 years with American Society of Anesthesiologists Physical Status (ASA PS) I, II, and III posted for knee arthroscopic anterior cruciate ligament repair surgeries. Patients with abnormal coagulation profiles, a history of allergy to local anesthetics, opioid and alcohol abuse, a history of psychiatric conditions, and a body mass index (BMI) >40 kg/m^2^ were excluded from the study. Randomization was done by a computer-generated randomization list, and participants were allocated to either of the two groups. Concealment was done using sealed opaque envelopes. Blinding was not possible but the data were collected by a person blinded to the intervention.

Group P (n = 30) received FNB with 20 mL 0.5% bupivacaine under the guidance of landmark with PNS. Group U (n = 30) received FNB with 20 mL 0.5% bupivacaine using USG.

Patients posted for arthroscopic procedures were prepared and the surgery was performed under a subarachnoid block using 2.5 mL of 0.5% bupivacaine.

Post-surgery, patients were shifted to the postoperative room, and the femoral block was given according to the computer-generated randomization list after two-segment regression of the sensory block.

In Group P, patients were given PNS-guided femoral block. The stimulation frequency was set at 1 Hz and the intensity of the stimulating current was initially set to deliver 1 mA. The electrode was attached to the anterior thigh. The needle was made air-free with the given drug, and under all aseptic conditions, the femoral artery was palpated on the operated limb just below the midpoint of the inguinal crease, and about 1.5-2 cm lateral to it, the point of insertion was marked. Using a 22-gauge insulated Stimuplex needle (B. Braun, Germany), the skin was punctured and the needle was manipulated and repositioned to elicit contraction of the quadriceps femoris muscle [7,8]. The current was then decreased slowly and the position of the needle was adjusted finely to get a negative stimulation at <0.3 mA. At that point, after negative aspiration for blood, the prepared solution was slowly injected.

In Group U, a 12-7 Hz high-frequency linear array probe (Fujifilm Sonosite M-Turbo Portable Ultrasound Machine) was used to locate the femoral artery. The needle insertion was done and was placed below the fascia iliaca, adjacent to the femoral nerve [6,9]. The needle placement was satisfactory if there was an expansion of the tissue below the fascia iliaca on an injection of 3 mL of the local anesthetic solution. If there was a poor distribution of the local anesthetic spread (spread into the more superficial structures or not visualized), then the needle was adjusted until the correct spread of local anesthetic was demonstrated, including spread to the anterolateral area of the femoral nerve [10]. After negative aspiration, the desired local anesthetic was injected over one to two minutes. Intravenous (IV) paracetamol (15 mg/kg every six hours) was administered to all patients. Injection tramadol 2 mg/kg IV was administered as a rescue analgesic if the patient complained of pain.

Time to perform the block (defined as the time from first needle contact with the skin to the removal of the needle), number of needle redirections required to achieve a block, and duration of the block (defined as the time from the deposition of the drug to the time after which the patient complained of pain and required a rescue analgesic) were noted. We also assessed patient response to overall satisfaction with the block as follows: 0: unsatisfactory, 1: satisfactory, 2: very good, or 3: outstanding [6].

Considering the mean difference and effect size based on a previous study by Marhofer et al. [9] with 80% power and 5% alpha error, 29 patients were required in each study arm. Patients were divided randomly into two groups of 30 with a sample size of 60 patients. For continuous variables, the data were presented as mean ± standard deviation (SD). The categorical variables were presented as frequency and percentage. The chi‑square or Fisher exact test was used to check the association between two categorical variables. Student’s t-test was used to test the significance of the difference between the two groups. Statistical analysis was done using the SPSS software version 20.0 (IBM Corp., Armonk, NY, USA), and p-values ≤0.05 were considered statistically significant.

## Results

**Figure 1:**
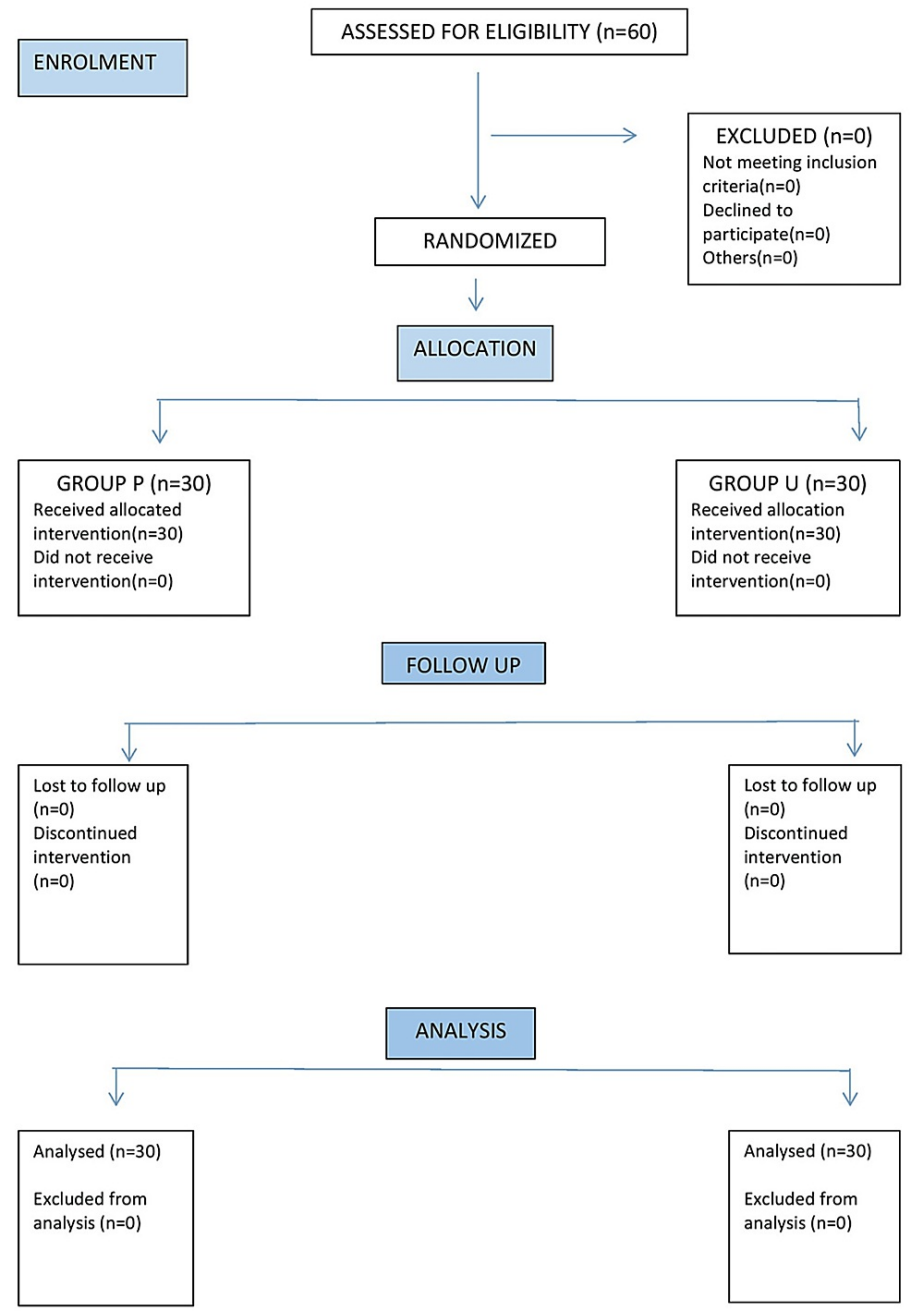
Consort diagram.

The study was conducted among a total of 60 patients divided into two groups of 30 each (Figure 1). The demographic data such as age, height, weight, BMI, and duration of surgery were comparable among the two groups (Table 1). There was a significant decrease in the number of repositioning in group U (2.57 ± 0.568) compared to group P (6.63 ± 0.718), as shown in Table 2. The mean time required to finish the procedure in group PNS was 11.20 ± 0.805 minutes, and the mean time required to finish the procedure in group U was 5.60 ± 0. 621 minutes (p < 0.001). There was a significant decrease in the time taken to finish the procedure with group U (Table 3). As shown in Table 4, there was no significant time difference between the two groups with respect to the duration of the effect of the block (p = 0.584). There was no significance between both groups with respect to patient satisfaction (p = 0.310) (Table 5).

**Table 1:** Demographic data.

Variables	Group P	Group U	P-value
Age (years)	28.57 ± 7.333	29.20 ± 7.232	0.737
Height (cm)	167.47 ± 4.075	167.53 ± 4.761	0.954
Weight (kg)	72.63 ± 4.694	72.00 ± 6.903	0.679
Body mass index (kg/m^2^)	25.87 ± 0.937	25.55 ± 1.298	0.289
Duration of surgery (minutes)	118.10 ± 5.647	117.40 ± 4.048	0.583

**Table 2:** Number of needle repositioning.

Group	Mean	Standard deviation	P-value
P	6.63	0.718	<0.001
U	2.57	0.568	

**Table 3:** Time taken to finish the procedure (in minutes).

Group	Mean	Standard deviation	P-value
P	11.20	0.805	<0.001
U	5.60	0.621	

**Table 4:** Duration of effect of the block (hours).

Group	Mean	Standard deviation	P-value
P	8.10	0.607	0.584
U	8.00	0.788	

**Table 5:** Patient satisfaction.

Group	Mean	Standard deviation	P-value
P	2.43	0.504	0.310
U	2.57	0.504	

## Discussion

To achieve an adequate block, we need to be able to locate the nerves accurately and deliver an appropriate dose of local anesthetics. USG reduces the duration of the technique, reduces the structural damage to the neurovascular bundle, and improves the success of the block.

Forouzan et al. [11] observed that USG-guided FNB had significantly lower procedural time compared to PNS, a lower need for rescue doses of opioids, and the block duration was almost similar in both groups. This was similar to our study with respect to the procedural time and the duration of the effect of the block.

Casati et al. [12] compared USG and PNS for multiple injection axillary brachial plexus block. They observed that the number of needle passes was four in the USG group compared to eight in the PNS group (p = 0.002), which was similar to our study, where we observed a lower number of needle repositioning in the USG group. They concluded that multiple injection axillary blocks with USG guidance provided similar success rates, which was consistent with our study.

Clinical trials performed by Perlas et al. and Rubin et al. in sciatic nerve block also supported the results of our study [13,14]. Kumar et al. [15] compared USG and PNS for multiple injection axillary brachial plexus block. They observed the number of skin punctures was two in the USG group and three in the PNS group (p < 0.001). This study correlates with our study where we observed that the only significant parameters were a decrease in the number of needle repositioning and time taken to perform the procedure in the group where USG was used. They concluded that multiple injection blocks with USG provide similar success and comparable incidence of complications with PNS, which was similar to our study.

We have not studied the postoperative opioid requirement in this study. Though data collection was done by a person blinded to the groups, blinding was not possible while performing the blocks which is an obvious limitation of our study.

## Conclusions

Based on the current trial, in experienced hands, USG and PNS-guided femoral block has the same success rate and block duration. However, USG significantly decreases the procedural time and the number of needle repositioning.
